# Supplementary material for: Characteristics of Cancer Patients in the World Trade Center Environmental Health Center
Source: Int J Environ Res Public Health. 2020 Oct 1;17(19):7190. doi: 10.3390/ijerph17197190 (PMC7578998; doi:10.3390/ijerph17197190)
Supplement: Supplementary file 1 [file ijerph-17-07190-s001.pdf]

Table S1: The list of cancers included in the WTC Pan-cancer database.

Cancer Type
Ampulla of vater
Anal canal
Bone
Brain and spinal cord
Breast
Cervix uteri
Colon and rectum
Connective and soft tissue
Corpus uteri
Esophagus
Extrahepatic bile duct
Fallopian tube
Gallbladder
Testis
Ovary
Head and neck sites
Kidney
Leukemia
Liver
Lung
Lymphoma
Melanoma of the skin
Mesothelioma
Myelodysplastic syndrome
Myeloma
Myeloproliferative neoplasm
Neuroendocrine tumor
Ophthalmic site tumor
Pancreas
Penis
Peritoneum/retroperitoneum
Prostate
Small Intestine
Soft tissue sarcoma
Stomach
Thymus
Thyroid
Urethra
Urinary bladder
Vagina
Vulva

Table S2: The median age of diagnosis and median latency from 9/11 for non-common cancers in both sexes.

	All (n= 2999)			Male (n= 1623)			Female (n= 1376)		
Cancer type	n (%)	Median age of diagnosis (range) (year)	Median latency (range) (year)	n (%)	Median age of diagnosis (range) (year)	Median latency (range) (year)	n (%)	Median age of diagnosis (range) (year)	Median latency (range) (year)
Liver	28 (1)	62 (48, 78)	13.3 (8, 17.8)	19 (1)	62 (49, 78)	13.7 (8, 17.5)	9 (1)	62 (48, 68)	12.9 (11.4, 17.8)
Brain and spinal cord	26 (1)	57 (36, 81)	14 (1.3, 17.8)	16 (1)	60 (36, 81)	14.2 (8, 17.8)	10 (1)	56 (36, 81)	12.8 (1.3, 17.6)

Esophagus	24 (1)	57 (32, 72)	12.2 (4, 16.6)	21 (1)	58 (32, 72)	12.7 (4, 16.6)	3 (0)	51 (47, 65)	11.2 (8.8, 11.8)
Myeloproliferative neoplasm	19 (1)	60 (5, 80)	13.8 (1.3, 16.6)	15 (1)	62 (5, 80)	13.8 (1.3, 16.6)	4 (0)	53.5 (45, 80)	10.1 (3.2, 16.5)
Small Intestine	18 (1)	57.5 (38, 78)	13.2 (5.4, 16.7)	10 (1)	58 (38, 72)	14 (7.8, 16.5)	8 (1)	54 (47, 78)	12.3 (5.4, 16.7)
Soft tissue sarcoma	16 (1)	53.5 (29, 75)	12.2 (6.3, 16.4)	11 (1)	53.5 (29, 75)	10.8 (6.3, 16.4)	5 (0)	52.5 (31, 58)	14.5 (10.2, 14.7)
Myelodysplastic syndrome	14 (0)	57 (43, 86)	13.8 (1.9, 16.4)	10 (1)	65 (53, 86)	14.3 (1.9, 16.4)	4 (0)	49 (43, 58)	12.8 (7.9, 14.3)
Corpus uteri	10 (0)	61.5 (52, 72)	14.1 (5.2, 16.9)				10 (1)	61.5 (52, 72)	14.1 (5.2, 16.9)
Extrahepatic bile duct	7 (0)	65.5 (54, 72)	14.8 (9.2, 16.8)	3 (0)	67 (64, 72)	15.2 (14.8, 16.8)	4 (0)	61 (54, 70)	13.2 (9.2, 14.8)
Cervix uteri	6 (0)	48.5 (44, 59)	12.1 (5.7, 16.6)				6 (0)	48.5 (44, 59)	12.1 (5.7, 16.6)
Thymus	6 (0)	57 (43, 71)	13.7 (12.2, 15.1)	5 (0)	58 (43, 71)	13.2 (12.2, 15.1)	1 (0)	57 (57, 57)	15 (15, 15)
Bone	5 (0)	56.5 (33, 73)	13.2 (9.2, 15.4)	4 (0)	59 (54, 73)	13.5 (9.2, 15.4)	1 (0)	33 (33, 33)	12.8 (12.8, 12.8)
Ophthalmic site tumor	4 (0)	54 (36, 69)	13.3 (5.3, 16.2)	2 (0)	54 (49, 59)	13.3 (11, 15.6)	2 (0)	52.5 (36, 69)	10.8 (5.3, 16.2)
Peritoneum/retroperitoneum	4 (0)	62 (50, 69)					4 (0)	62 (50, 69)	9.8 (8.0, 16.0)
Ampulla of vater	3 (0)	63 (62, 76)	14.9 (13.2, 15.5)	2 (0)	69 (62, 76)	14.3 (13.2, 15.5)	1 (0)	63 (63, 63)	14.9 (14.9, 14.9)
Anal canal	3 (0)	67 (43, 68)	14.2 (9.7, 15.8)	1 (0)	67 (67, 67)	9.7 (9.7, 9.7)	2 (0)	55.5 (43, 68)	15 (14.2, 15.8)
Fallopian tube	3 (0)	59 (52, 63)	15 (13.6, 16.9)				3 (0)	59 (52, 63)	15 (13.6, 16.9)
Penis	3 (0)	56 (55, 61)	15.4 (15, 16.7)	3 (0)	56 (55, 61)	16 (15.4, 16.7)			15 (15, 15)
Vulva	3 (0)	71 (59, 77)	13.5 (12.1, 16)			13.5 (12.1, 16)	3 (0)	71 (59, 77)	
Unknown primary site	3 (0)	50 (39, 69)	15.2 (14.9, 15.8)	2 (0)	54.5 (40, 69)		7 (1)	50 (39, 69)	15.2 (14.9, 15.8)
Gallbladder	2 (0)	65.5 (61, 70)	13.1 (12, 14.2)			14.2 (14.2, 14.2)	2 (0)	65.5 (61, 70)	12 (12, 12)
Connective and soft tissue	2 (0)	39.5 (39, 40)	11.2 (9.7, 12.7)	1 (0)	40 (40, 40)		1 (0)	39 (39, 39)	11.2 (9.7, 12.7)
Neuroendocrine tumor	1 (0)	63 (63, 63)	13.6 (11.1, 16.1)	1 (0)	63 (63, 63)	11.1 (11.1, 11.1)			16.1 (16.1, 16.1)
Urethra	1 (0)	72 (72, 72)	15.3 (15.3, 15.3)	1 (0)	72 (72, 72)	15.3 (15.3, 15.3)			
Vagina	1 (0)	66 (66, 66)	13.2 (13.2, 13.2)			13.2 (13.2, 13.2)	1 (0)	66 (66, 66)	
			14 (14, 14)						14 (14, 14)
